# Tracking single units in chronic, large scale, neural recordings for brain machine interface applications

**DOI:** 10.3389/fneng.2014.00023

**Published:** 2014-07-08

**Authors:** Ahmed Eleryan, Mukta Vaidya, Joshua Southerland, Islam S. Badreldin, Karthikeyan Balasubramanian, Andrew H. Fagg, Nicholas Hatsopoulos, Karim Oweiss

**Affiliations:** ^1^Department of Electrical and Computer Engineering, Michigan State UniversityEast Lansing, MI, USA; ^2^Department of Organismal Biology and Anatomy, Committee of Computational Neuroscience, University of ChicagoChicago, IL, USA; ^3^Department of Computer Science and Bioengineering, University of OklahomaNorman, OK, USA; ^4^Neuroscience Program, Michigan State UniversityEast Lansing, MI, USA; ^5^Cognitive Science Program, Michigan State UniversityEast Lansing, MI, USA

**Keywords:** single-units, stability, BMI, waveforms distance, ISIH distance

## Abstract

In the study of population coding in neurobiological systems, tracking unit identity may be critical to assess possible changes in the coding properties of neuronal constituents over prolonged periods of time. Ensuring unit stability is even more critical for reliable neural decoding of motor variables in intra-cortically controlled brain-machine interfaces (BMIs). Variability in intrinsic spike patterns, tuning characteristics, and single-unit identity over chronic use is a major challenge to maintaining this stability, requiring frequent daily calibration of neural decoders in BMI sessions by an experienced human operator. Here, we report on a unit-stability tracking algorithm that efficiently and autonomously identifies putative single-units that are stable across many sessions using a relatively short duration recording interval at the start of each session. The algorithm first builds a database of features extracted from units' average spike waveforms and firing patterns across many days of recording. It then uses these features to decide whether spike occurrences on the same channel on one day belong to the same unit recorded on another day or not. We assessed the overall performance of the algorithm for different choices of features and classifiers trained using human expert judgment, and quantified it as a function of accuracy and execution time. Overall, we found a trade-off between accuracy and execution time with increasing data volumes from chronically implanted rhesus macaques, with an average of 12 s processing time per channel at ~90% classification accuracy. Furthermore, 77% of the resulting putative single-units matched those tracked by human experts. These results demonstrate that over the span of a few months of recordings, automated unit tracking can be performed with high accuracy and used to streamline the calibration phase during BMI sessions. Our findings may be useful to the study of population coding during learning, and to improve the reliability of BMI systems and accelerate their deployment in clinical applications.

## 1. Introduction

Invasive Brain Machine Interfaces (BMIs) for neuro-motor prosthetics rely on neural signals recorded using chronically implanted microelectrodes to actuate external devices (Serruya et al., 2002; Kim et al., 2008; Velliste et al., 2008; Suminski et al., 2010; Hochberg et al., 2012). In this setting, signal and information stability play a crucial role in maintaining a clinically viable BMI that subjects can use routinely in their home environment. Recent evidence suggest that within-day fluctuations in action potential amplitude and inter-spike intervals recorded with microelectrode arrays implanted in humans could result in a directional “bias” in the decoded neural activity used to actuate an artificial device; and this results in suboptimal performance (Perge et al., 2013). Other studies have shown that when subjects are repeatedly exposed to a neural decoder presumably driven by the same population across days, they build a stable map of cortical responses that “fit” that particular decoder (Ganguly and Carmena, 2009, 2010; Koyama et al., 2010). As such, a fixed decoder model paired with stable neural inputs may permit the consolidation of the motor memory of that particular decoder during BMI practice. Stability of neural recordings with penetrating microelectrodes, however, is hard to maintain due to biotic and abiotic factors beyond the experimenter's control (Karumbaiah et al., 2013). As such, state of the art BMIs lack reliability and require human operators to frequently identify units and to train new decoders on a daily basis- a process that can be viewed as *interference* to motor-memory consolidation, which diminishes the ability to retain motor skills and reduces BMI robustness.

The standard approach to mitigate this instability is to rely on a human operator to ascertain that units intermittently recorded on previous days remain the same on the current day *before* the start of a BMI session. When changes do occur, the operator has to select new populations and perhaps train a new decoder by collecting a few minutes of neural data while subjects are asked to imagine or attempt limb movements, or observe these movements from previous training sessions (Hochberg et al., 2012; Homer et al., 2013). A fast, automatic, and efficient approach to assess stability of these putative single-units at the start of every session would be highly desirable to streamline the calibration phase without much intervention from the human operator and without imposing additional cognitive load on the subject. It could also be beneficial in basic neuroscience investigations of learning and memory formation at the population level (Buzsáki, 2004).

In this study, we propose a fast and accurate algorithm to assess unit stability across days with minimal human expert intervention. We tested the performance of this algorithm on data collected using fixed silicon microelectrode arrays chronically implanted in primary motor cortex (M1) of Rhesus Macaques (*Macaca mulatta*) over many months. The performance was benchmarked against that of human experts who manually labeled the data to provide practical ground truth.

## 2. Materials and methods

### 2.1. Algorithm overview

**Figure 1:**
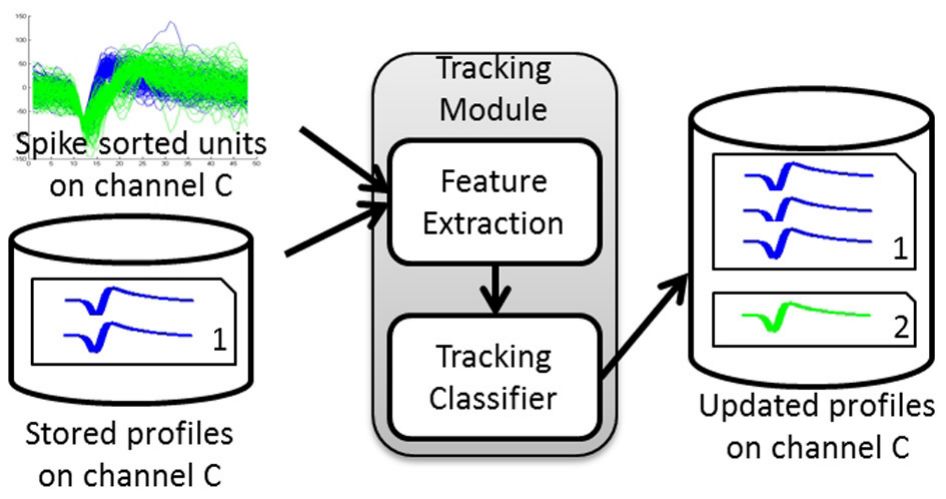
**Tracking Algorithm overview**. The tracking module takes as input the spike-sorted single unit discharge patterns and the stored profiles for a given channel and calculates the dissimilarity between these units' features and the stored profiles. It then assigns each unit to either an existing profile or a new profile based on the dissimilarity computed. The output is the updated set of profiles for that channel.

An overview of the algorithm steps is shown in Figure 1. The algorithm first builds a database of profiles of putative single-units recorded across a multi-day interval set by the user. Each putative single-unit profile (hereafter referred to as a profile for simplicity) consists of a collection of spike occurrences from this putative single-unit on each day. Such collection contains user-defined information extracted from the single-unit activity, such as average waveform, spike timestamps, inter-spike interval histogram (ISIH), firing rate, etc.

The tracking module has two main blocks: (1) feature extraction and (2) tracking classifier. The inputs to the tracking module are the spike-sorted waveforms recorded on any given channel. The module extracts features from these inputs, retrieves the stored single-unit profiles for each channel, and finds the pairwise dissimilarities between the input features and those in the stored profiles. The dissimilarity vectors are then passed to the tracking classifier that makes decisions on whether to add an input unit to an existing profile (match) or to create a new profile (non-match). The output of the tracking module is then a set of updated profiles for each recording channel.

### 2.2. Single-unit waveform characteristics

We selected four features to extract from each average spike waveform in a unit's profile. We also selected four dissimilarity measures to quantify the difference between features from two average spike waveforms, W_*x*_ and W_*y*_, recorded from putative single-units “*x*” and “*y*” on the same channel on different days. The average waveform for unit *x*, W_*x*_, is calculated as:

(1) $${\bar{\text{W}}}_{x}=\left[\sum_{i=1}^{l}\frac{{\text{W}}_{x}^{i}(1)}{l}\ldots \sum_{i=1}^{l}\frac{{\text{W}}_{x}^{i}(m)}{l}\right]\text{​},$$

where *l* is the number of sample waveforms assigned to a single unit *x* during spike sorting, and *m* is the number of samples in the waveform.

Table 1 lists the chosen measures and the waveform features that each of these measures relies upon to compute the dissimilarity.

**Table 1:** **Waveform-derived dissimilarity measures**.

**Dissimilarity measure**	**Feature**
Correlation coefficient	Waveform shape
Normalized peak-to-peak height difference	Amplitude
Normalized peak-to-peak time difference	Transition time between minimum and maximum peaks
Peak matching	Peaks shape

**Pearson correlation coefficient** (PC) (Nikolić et al., 2012) was used to compare the shape similarity of the waveforms. The **normalized peak-to-peak height difference** (PH) was used to quantify the difference in the amplitude of the two waveforms and to normalize it to the amplitude of one of them as:

(2) $$PH\left({\bar{\text{W}}}_{x},{\bar{\text{W}}}_{y}\right)=\left|\frac{\left(\max\left({\bar{\text{W}}}_{y}\right)-\min\left({\bar{\text{W}}}_{y}\right)\right)-\left(\max\left({\bar{\text{W}}}_{x}\right)-\min\left({\bar{\text{W}}}_{x}\right)\right)}{\left(\max\left({\bar{\text{W}}}_{y}\right)-\min\left({\bar{\text{W}}}_{y}\right)\right)}\right|,$$

where max and min are the waveform maximum and minimum values, respectively. Therefore, a difference between the peak-to-peak values of two high-amplitude units would result in a smaller PH value than if the same difference was calculated between two low-amplitude units. This is desirable in order to reflect that a large difference between two low amplitude units indicates higher variability than the case of two high amplitude units. The **normalized peak-to-peak time difference** (PT) was used to find the difference in the transition time between the minimum and maximum peaks of the waveform and was defined as:

(3) $$PT\left({\bar{\text{W}}}_{x},{\bar{\text{W}}}_{y}\right)=\left|\frac{\begin{array}{c} \left(\underset{i}{\mathrm{argmax}}\left({\bar{\text{W}}}_{y}(i)\right)\text{​}-\text{​}\underset{i}{\mathrm{argmin}}\left({\bar{\text{W}}}_{y}(i)\right)\right)\\ \;-\left(\underset{i}{\mathrm{argmax}}\left({\bar{\text{W}}}_{x}(i)\right)\text{​}-\text{​}\underset{i}{\mathrm{argmin}}\left({\bar{\text{W}}}_{x}(i)\right)\right) \end{array}}{\begin{array}{c} \left(\underset{i}{\mathrm{argmax}}\left({\bar{\text{W}}}_{y}(i)\right)\text{​}-\text{​}\underset{i}{\mathrm{argmin}}\left({\bar{\text{W}}}_{y}(i)\right)\right) \end{array}}\right|,$$

where $\underset{i}{\text{argmax}}$ and $\underset{i}{\text{argmin}}$ are operators that retrieve the sample number *i* (i.e., time index) corresponding to the waveform maximum and minimum, respectively.

Combined with PH, we are essentially comparing the slopes of the lines joining the minimum and maximum peaks of the two waveforms. The **Peak Matching** (PM) is a measure developed by Strelkov (2008) that heuristically computes the distance between the shapes of the peaks of two signals. The algorithm finds the peaks in both signals and assigns weights to each peak. It then finds pairwise closeness factors (i.e., similarity measures) between these peaks and finally calculates the total distance as the sum of peak weights multiplied by the computed closeness factors. Details of the calculation of each entity can be found in Strelkov (2008). The symmetrized similarity between the two signals is calculated as the geometric mean of the asymmetric similarities.

### 2.3. Single-unit firing characteristics

Assuming negligible change in unit recruitment properties, differences in firing patterns can be used as a metric to aid in unit stability assessment (Liu et al., 1999; Chen and Fetz, 2005). These differences can be quantified by computing the dissimilarity between normalized ISIHs H_*x*_ and H_*y*_ (i.e., empirical probability distributions of inter-spike intervals). We chose four such dissimilarity measures to assess multiple aspects of the difference between two probability distributions. Table 2 lists the chosen measures and the criterion that each of them uses to compute the dissimilarity. The merit of each dissimilarity measure is explained below.

**Table 2:** **ISIH-derived dissimilarity measures**.

**Dissimilarity measure**	**Calculation**	**Dissimilarity criterion**
Symmetrized KL-divergence	$\begin{array}{l} D({\text{H}}_{x}||{\text{H}}_{y})=\sum_{i=1}^{l}\ln\left(\frac{{\text{H}}_{x}[i]}{{\text{H}}_{y}[i]}\right){\text{H}}_{x}[i]\\ D({\text{H}}_{x},{\text{H}}_{y})=\frac{D({\text{H}}_{x}||{\text{H}}_{y})+D({\text{H}}_{y}||{\text{H}}_{x})}{2} \end{array}$	Total information divergence
Bhattacharyya distance	$BD({\text{H}}_{x},{\text{H}}_{y})=-\ln\sum_{i=1}^{l}\sqrt{{\text{H}}_{x}[i]{\text{H}}_{y}[i]}$	Amount of overlap
KS-statistic	$\begin{array}{l} \text{ F}[i]=\sum_{k=1}^{i}\text{H}[k]\\ KS({\text{H}}_{x},{\text{H}}_{y})=\underset{{}_{i=1\ldots l}}{\max}\left|{\text{F}}_{x}[i]-{\text{F}}_{y}[i]\right| \end{array}$	Maximum divergence
Earth mover's distance	$\begin{array}{l} EMD_{0}=0\\ EMD_{i+1}=({\text{H}}_{x}[i]+EMD_{i})-{\text{H}}_{y}[i]\\ EMD({\text{H}}_{x},{\text{H}}_{y})=\sum_{i=1}^{l}\left|EMD_{i}\right| \end{array}$	Cost of turning one distribution to the other

It should be noted that while the Kullback-Leibler Divergence (KLD) (Kullback and Leibler, 1951; Johnson and Sinanovic, 2001) calculates the total divergence between two distributions, the Kolmogorov-Smirnov Statistic (KS) (Gürel and Mehring, 2012) approximates that divergence by including the maximum difference at any point in the cumulative distributions. Both the KLD and the KS provide different perspectives when comparing two distributions. The former measure overlooks individual points and is only concerned with the total divergence across all the points, while the latter measure does not. The Bhattacharyya Distance (BD) (Bhattacharyya, 1943) quantifies the amount of overlap between the two distributions to decide how similar they are, while the Earth Mover's Distance (EMD) (Rubner et al., 1998) computes the minimum cost of converting one distribution to the other.

### 2.4. The tracking classifier

Given a vector of dissimilarities between the features extracted from two single units recorded on the same channel on two different days, the tracking classifier makes the binary decision of whether these two single-units represent different instances of the same single unit or not. Training a classifier requires supervision and for this purpose two sets were defined:

- **True positives (TP)** are vectors of dissimilarities between pairs of instances recorded from the same neuron across a number of days. They were obtained by manual tracking performed by two experts on the recorded data on those days.
- **True negatives (TN)** are vectors of dissimilarities between pairs of instances recorded from different neurons. They were obtained using different units recorded on the same channel on a given day. Units recorded simultaneously on the same channel on a given day are different units.

**Figure 2:**
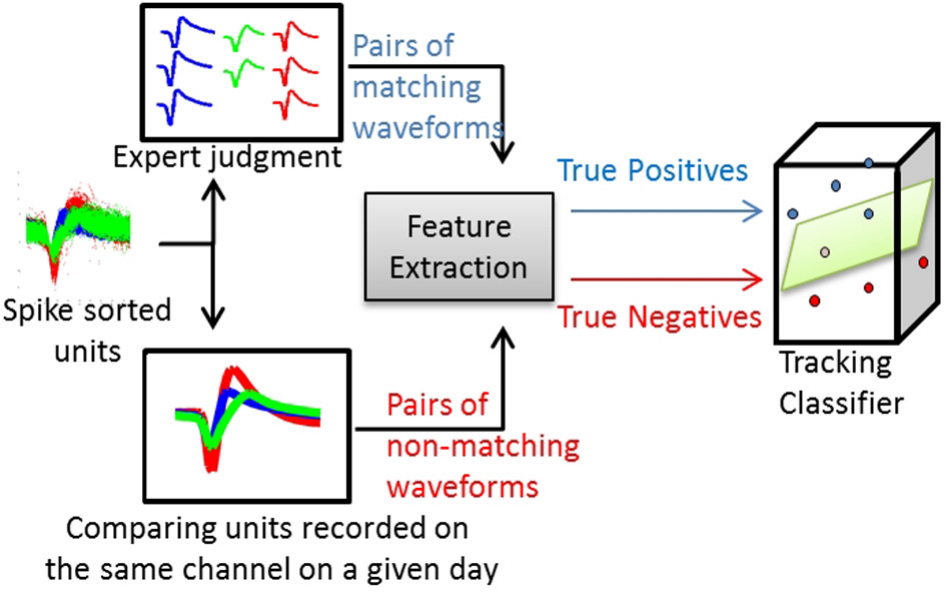
**Process of training a waveform-based classifier**. Using spike-sorted units recorded across several days, human experts track the units manually and produce pairs of matching average waveforms (true positives). Using the same set of units, pairs of non-matching average waveforms are generated by comparing spike-sorted units recorded simultaneously on the same channel on a given day.

For the waveform-based classifier, true positives and true negatives were computed by comparing the average waveforms. Figure 2 illustrates this process. The same process was applied for training classifiers that used other single-unit characteristics (e.g., ISIHs).

The input to a classifier was a vector, *x*, of the dissimilarities between the features extracted from two units recorded on the same channel but on different days. The length, *n*, of the dissimilarities vector is the number of dissimilarity measures used in the comparison. The classifiers' decision is binary, either to assign *x* to class 0 (*c*_0_), which corresponds to non-matching units, or to class 1 (*c*_1_), which corresponds to matching units. Several types of classifiers were compared in this study. The following section provides a brief summary of the classifiers used.

#### 2.4.1. Support vector machine (SVM) classifier

The SVM classifier is a binary classifier that finds a high-dimensional decision plane. This plane is inferred so as to maximize the distance between the plane and the training data points of each class. The SVM model takes the form (Bishop, 2006)

(4) $$\text{y}(\text{x})={\text{w}}^{\text{T}}\phi (\text{x})+\text{b},$$

where, w defines the decision plane, x is the input dissimilarity vector, ϕ(x) is a projection of the input onto the feature space (basis function), and b is an offset term to account for any bias in the training data.

Although the SVM model in Equation (4) is linear, non-linearity can be added to the model by the use of a non-linear basis function (known as kernels) such a Gaussian radial basis function (Bishop, 2006).

#### 2.4.2. Relevance vector machine (RVM) classifier

The RVM calculates the classification probability rather than the classification decision as in the case of the SVM. The model then becomes of the form (Bishop, 2006)

(5) $$\text{y}(\text{x})=\sigma \left({\text{w}}^{\text{T}}\phi (\text{x})+\text{b}\right),$$

where σ(.) is the sigmoid function.

#### 2.4.3. Maximum a posteriori classification (MAP)

The MAP is a probabilistic classifier and is used to estimate how likely a new point belongs to a distribution based on empirical data.

Given the dissimilarity vector between two units, *x* = [*x*_1_, …, *x_n_*]^*T*^, the MAP classification rule takes the form (Bishop, 2006):

(6) $$p(x_{1},\ldots ,x_{n}|c_{1})P(c_{1})\underset{c_{0}}{\overset{c_{1}}{≷}}p(x_{1},\ldots ,x_{n}|c_{0})P(c_{0}),$$

where *p*(*x*_1_, …, *x_n_*|*c*_0_) and *p*(*x*_1_, …, *x_n_*|*c*_1_) are the likelihood probabilities that *x* belongs to *c*_0_ and *c*_1_, respectively, and *P*(*c*_0_) and *P*(*c*_1_) are the prior probabilities of *c*_0_ and *c*_1_, respectively.

The likelihood and prior probability distributions are computed from the training data, where the true positives are instances of *c*_1_ and the true negatives are instances of *c*_0_.

#### 2.4.4. Naive bayesian classification (NB)

The NB is a simple and effective probabilistic classifier that, unlike the MAP, assumes independence between features (Domingos and Pazzani, 1997). Therefore, instead of using a joint conditional probability distribution as in the case of MAP, the NB uses multiple individual conditional probability distributions to infer its decision. For a dissimilarity vector between two units, *x*, the NB decision is (Manning et al., 2008):

(7) $$\prod_{i=1}^{n}p(x_{i}|c_{1})\underset{c_{0}}{\overset{c_{1}}{≷}}\prod_{i=1}^{n}p(x_{i}|c_{0}),$$

where *p*(*x_i_*|*c*_0_) and *p*(*x_i_*|*c*_1_) are the conditional probability distributions that the *i*^th^ feature of *x* belongs to *c*_0_ and *c*_1_, respectively.

As in the MAP, the conditional probability distributions are constructed using training data, where the true positives are instances of *c*_1_ and the true negatives are instances of *c*_0_.

### 2.5. Tracking algorithm

The average waveforms were smoothed using a low-pass filter with Gaussian kernel to reduce the high frequency components that result from the short recording duration. Each unit profile stores instances of the corresponding single unit recorded across days. On a given day, units on an arbitrary channel *c* are spike sorted and passed to the feature extraction module. Pairwise dissimilarity vectors are computed between the stored profiles on channel *c* and the input spike sorted units. A dissimilarity vector is the concatenation of the dissimilarity measures computed over an input unit and a stored profile instance.

**Figure 3:**
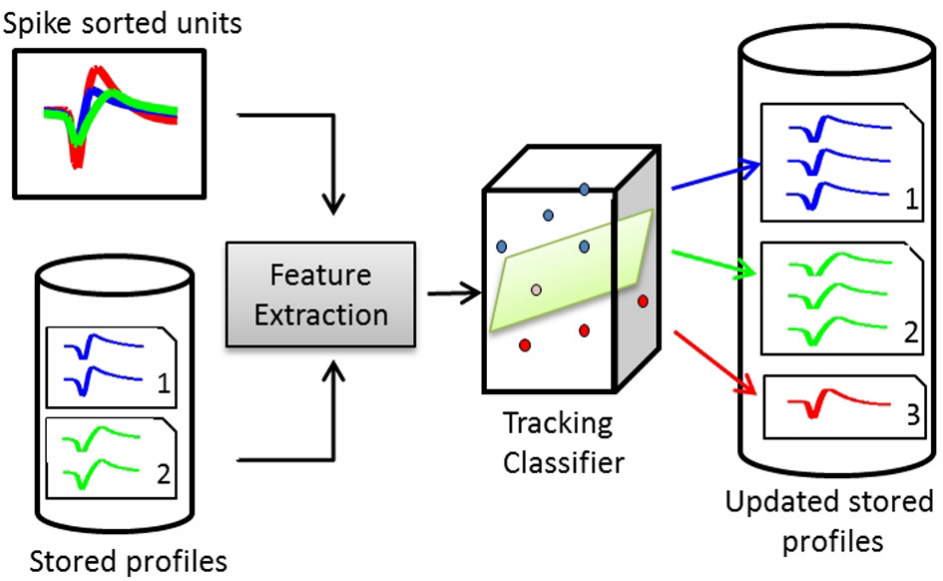
**Structure of a waveform-based classifier**. On day *x*, the spike-sorted units of channel *c* are compared to the stored profiles on the same channel. Pairwise dissimilarity vectors are calculated from features extracted from pairs of waveforms. The tracking classifier makes the decision on whether to add the units to existing profiles or create new profiles for them.

A dissimilarity matrix is built using the pairwise dissimilarity vectors between the stored profiles and the spike sorted units. The dissimilarity matrix is then input to the tracking classifier to build a membership matrix, where each cell indicates whether a single unit from an input is another instance of a stored profile. Figure 3 illustrates the structure of a waveform-based classifier.

Denote the set of profiles stored on channel *c* as *P*_c_, the set of spike sorted input units on channel *c* as *U_c_*, the dissimilarity between the *i*th profile and the *j*th unit as *Dis*(*P_c,i_, U_c,j_*), and the membership of the *j*th unit to the *i*th profile as *Mem*(*P_c,i_, U_c,j_*).

Assume there are *m* stored profiles on *c*, and *n* spike sorted units on day *x*. The dissimilarity matrix is formed as:

$$DisMat_{c}(P,U)=\left[\begin{array}{ccc} Dis(P_{c,1},U_{c,1}) & \ldots & Dis(P_{c,1},U_{c,n})\\ \vdots & \ddots & \vdots \\ Dis(P_{c,m},U_{c,1}) & \ldots & Dis(P_{c,m},U_{c,n}) \end{array}\right]$$

And the membership matrix is:

$$MemMat_{c}(P,U)=\left[\begin{array}{ccc} Mem(P_{c,1},U_{c,1}) & \ldots & Mem(P_{c,1},U_{c,n})\\ \vdots & \ddots & \vdots \\ Mem(P_{c,m},U_{c,1}) & \ldots & Mem(P_{c,m},U_{c,n}) \end{array}\right]$$

Finally, the relationship between the dissimilarity and membership matrices can be modeled as:

(8) $$DisMat_{c}(P,U)\Rightarrow \text{TrackingClassifier}\Rightarrow MemMat_{c}(P,U)$$

Units are added as instances of the profiles to which they belong. If a new unit is identified, a new profile is initialized and the unit instance is added to it. A profile is composed of all the recorded instances of the single unit it represents. When an input unit is compared to a profile to make the decision as to whether the unit is another instance of the profile, the tracking classifier decides which profile instance (i.e., from which day) to use in such comparison. We developed two techniques—the “**First Matching Dissimilarity (FMD)**” and the “**Maximum Matching Dissimilarity (MMD)**” that use a “stability window” of 7 days based on results in Dickey et al. (2009).

1. **First Matching Dissimilarity (FMD)**: In this technique, the most recent seven instances in a profile during the stability window are used in the comparison, from newest to oldest. Once the tracking classifier indicates that a profile instance and the unit are recorded from the same single unit (a match), the comparison stops and this instance is used for dissimilarity vector calculations.
2. **Maximum Matching Dissimilarity (MMD)**: In this technique, the comparison is performed based on all the profile instances that occurred within the stability window. The dissimilarity vector resulting from this comparison is the dissimilarity between the unit and the profile instance *closest* to it in terms of the distance to the decision boundary.

The intuition behind the two techniques is to tolerate units that undergo some minor instability for a short period of time, for example due to noise from the electronic circuitry used in recording on a given day, and then return to their stable state. We also defined two classes of profiles: *active* and *inactive*. Active profiles are those profiles whose single units have been recorded on any day within the stability window. Inactive profiles, on the other hand, are those profiles whose units did not appear in the recording on all days during the stability window, in which case we consider them “*dropped units*” and are not included in future comparisons.

Training each classifier results in a membership matrix, where each cell in this matrix indicates whether an input unit on a given day should be added to a stored profile. In some cases, the classifier might indicate that the input unit can be added to more than one profile, for example because of misclassifications in the spike sorting algorithm. A putative single-unit, however, can only be added to one profile. Therefore, we developed a back-tracking algorithm to find the best assignment of input single units to existing profiles. Back-tracking is a standard general algorithm for finding all the possible solutions to a problem and then selecting one that most satisfies a set of user-defined objectives (Cormen et al., 2001). In our case, the objectives were as follows:

- **Primary objective**: Maximize the number of assigned units to already existing stored profiles.
- **Secondary objective**: Maximize the total sum of distances to the decision boundary of the assigned units, since the larger the distance to the boundary, the more certain the decision of the tracking classifier is.

This way, the back-tracking algorithm finds the membership matrix MemMat^*^_c_(*P,U*) that meets both the primary and secondary objectives while assigning every input unit to one and only one database profile. This means that if two unit assignment solutions have the same number of units assigned to existing profiles, the one with a larger sum of distances to the decision boundary is selected. The relationship between the different data matrices can be modeled as:

(9) $$\begin{array}{l} DisMat_{c}(P,U)\Rightarrow \text{TrackingClassifier}\Rightarrow MemMat_{c}(P,U)\\ \;\Rightarrow \text{Backtracking}\Rightarrow MemMat_{c}^{*}(P,U) \end{array}$$

The tracking algorithm is summarized in Algorithm 1

**Algorithm 1:** **Tracking algorithm**.

**Input:** Signal recorded on all channels, database of stored single unit profiles
**Output:** Updated database of stored single unit profiles
**for** each channel *c* **do**
Spike sort the signal recorded on *c*
Input the spike sorted units to the tracking module
Compute distance vectors between each of the input units and the stored profiles associated with *c*
Using the distance vectors computed, build a membership matrix between the input units and the stored profiles on *c* using the tracking classifier
Using back-tracking, generate all possible assignments of the input units to existing profiles
Select the assignment that maximizes the number of assigned units to existing profiles
Create new profiles for unassigned units
**end for**

### 2.6. Data acquisition and behavioral task

Recordings were obtained using a 96-microelectrode silicon array (Blackrock Microsystems, Inc., Salt Lake City, UT) chronically implanted in the primary motor cortex (M1) of a rhesus macaque (Macaca mulatta). The first recording used in this study was made three months after the implant. The surgical and behavioral procedures of this study were approved by the University of Chicago Institutional Animal Care and Use Committee and conform to the principles outlined in the Guide for the Care and Use of Laboratory Animals. Spike sorting was done using the Cerebus® Online Sorter (Blackrock microsystems, UT) and the sorting templates were updated on a daily basis by a human operator.

The subject performed a brain-controlled reach-to-grasp task. Details of the behavioral task can be found in Balasubramanian et al. (2013). Fifteen datasets, spanning a period of 26 days, were used in training (seven datasets) and testing (eight datasets) the tracking algorithm. We used only the first 15 min of each dataset, which is equivalent to the period of updating the spike sorting templates while the subject is not engaged in any behavioral task. For execution time measurements, we used an Intel-i7 quad-core processor (3.40 GHz) and 16 GB of RAM.

### 2.7. Tracking algorithm evaluation

The performance of the tracking algorithm was assessed based on two metrics:

1. **Classification Accuracy**: The percentage of correct classification made on a day-to-day basis compared to manual tracking. A correct classification happens when a unit on day *x* is matched with the correct putative single-unit profile on day *x*−1.
2. **Percentage of Correct Profiles**: The percentage of profiles that were correctly tracked across all days of the testing datasets compared to manual tracking. A correct profile is a profile that has exactly the same single-unit instances as in the corresponding manually tracked profile across all days.

Classifier implementations were provided by the **newfolder library** (Torrione et al., 2011). We used a radial basis function as the kernel for the SVM and RVM classifiers where the Gaussian kernel sigma was set to the square root of the number of features. The slack variable of the SVM classifer was defaulted to 1. We compared the performance of the classifiers using four different sets of features:

- **Set 1**: 4 waveform-based features (PC, PH, PT, PM) and 4 ISIH-based features (KLD, BD, KS, EMD).
- **Set 2**: 3 waveform-based features (PH, PT, PM) and 3 ISIH-based features (KLD, BD, KS).
- **Set 3**: 3 ISIH-based features (KLD, BD, KS).
- **Set 4**: 3 waveform-based features (PH, PT, PM).

Performing validation was challenging because the calendar-day order of the datasets had to be maintained. This is because changes in the characteristics of a single unit might be small over two successive datasets (calendar-days) but much larger across datasets with longer time separation. We distributed the fifteen datasets (see section 2.6) into six validation datasets. Each validation dataset was divided into five training datasets and five testing datasets. The first validation dataset contained datasets 1–10, the second validation datasets contained datasets 2–11, and so on.

## 3. Results

### 3.1. Training features

**Figure 4:**
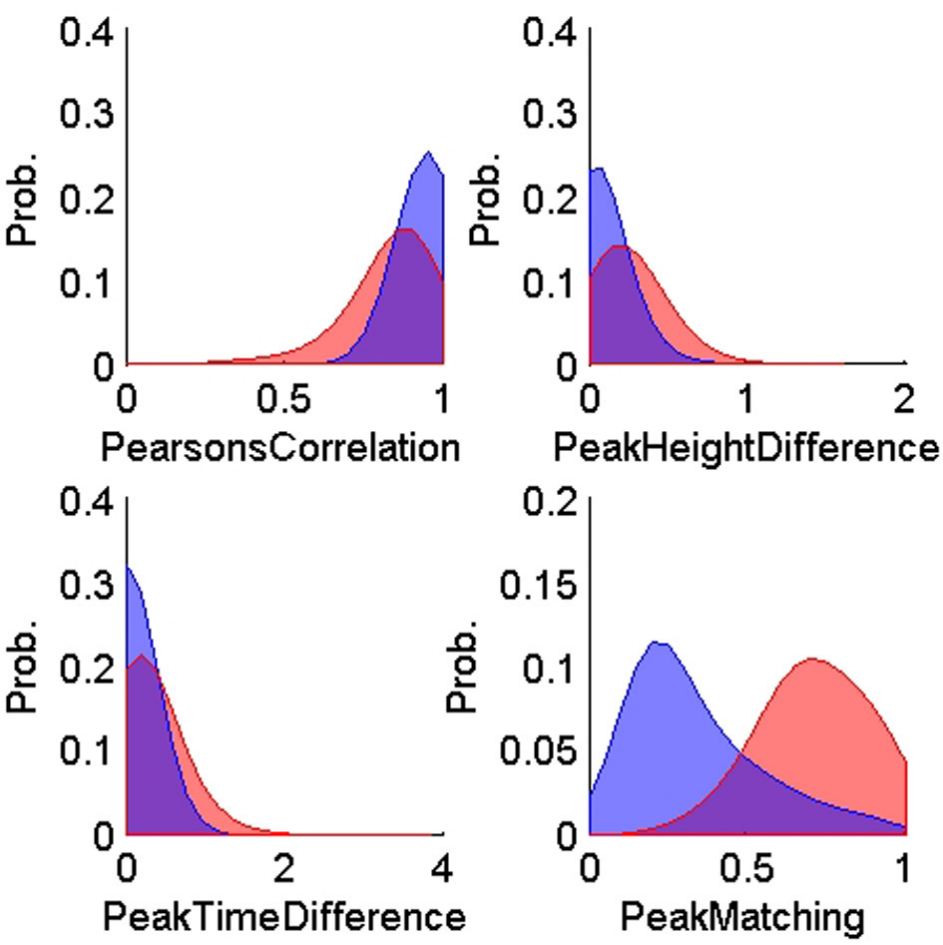
**Probability distributions of the training true positives (*n* = 900, blue) and true negatives (*n* = 600, red) for each of the four waveform-based dissimilarity measures: Pearson correlation's, peak-to-peak height difference, peak-to-peak time difference, and peak matching**. The training data was obtained using human expert judgment. The peak matching provided the best separability between matching and non-matching waveforms.

**Figure 5:**
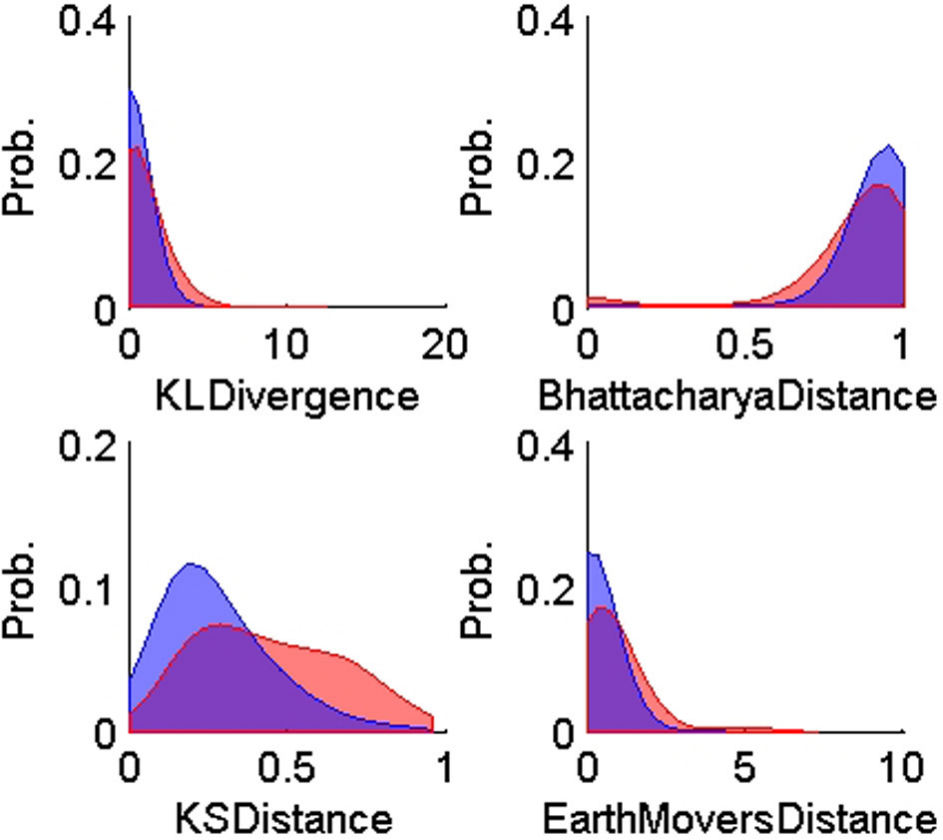
**Probability distributions of the training TP (*n* = 900, blue) and TN (*n* = 600, red) for each of the four ISIH-based dissimilarity measures: KL-divergence, Bhattacharya distance, KS-distance, and Earth Mover's distance**. The training data was obtained using human expert judgment. All ISIH-based dissimilarity measures did not provide enough separation between matching and non-matching ISIH.

**Figure 6:**
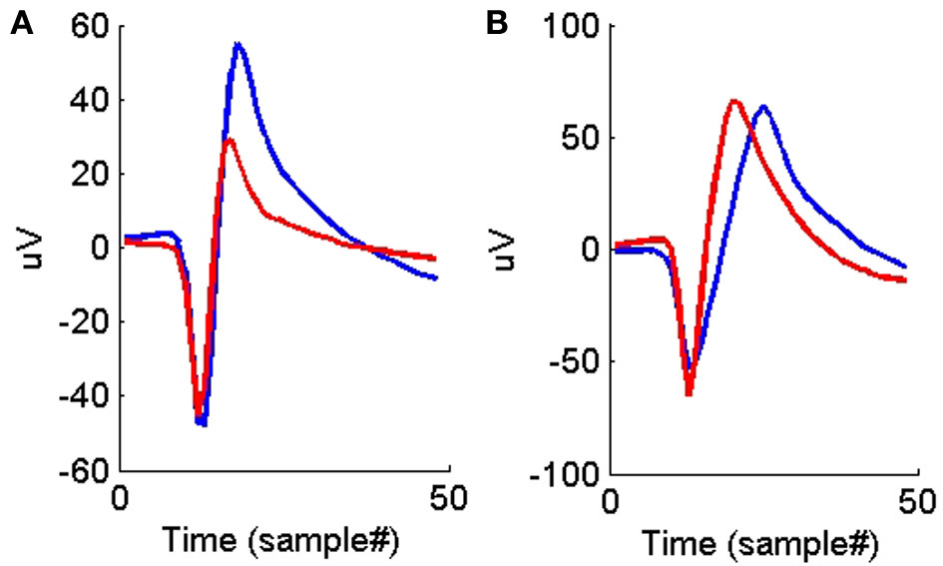
**(A)** An example of two average waveforms that have very similar peak shapes but different peak-to-peak heights. **(B)** An example of two average waveforms that have very similar peak shapes but different peak-to-peak times.

Figures 4, 5 show the distributions of the waveform- and ISIH- based dissimilarity measures applied to the TP and TN training datasets. We found a variable degree of separability between the distributions provided by each of the features selected. For example, while the peak matching distance provided the best separability between matching and non-matching waveforms, the other three waveform-based dissimilarity measures did not provide sufficient separation between classes, except in regions where it was very clear that there is a significant difference in the amplitude or time between peaks. The inclusion of the other features, however, was necessary to account for cases when two waveforms had very similar peak shapes, but significantly different amplitude- or time-between-peaks, which occurred frequently in our data (see Figure 6).

**Figure 7:**
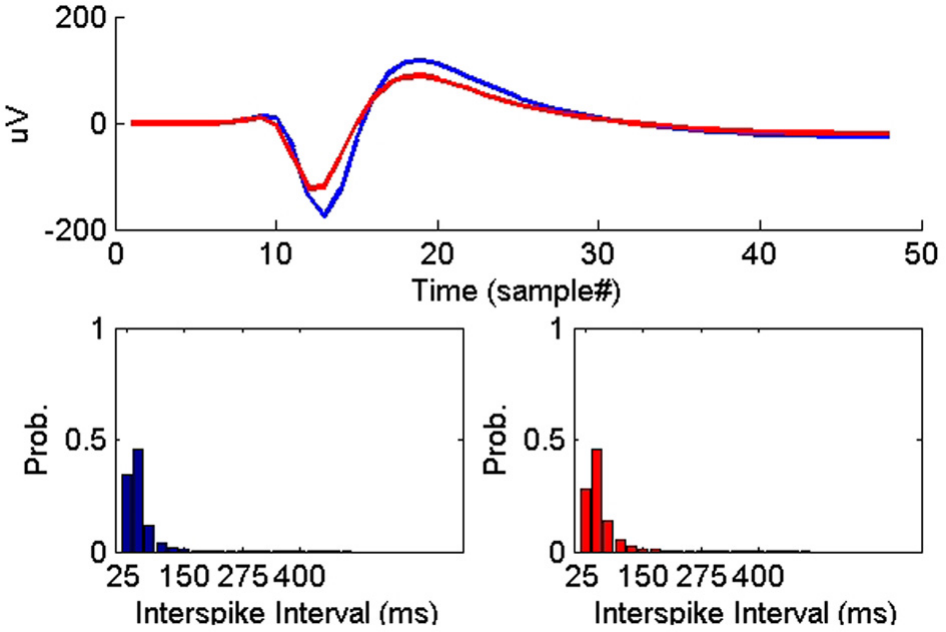
**Example of two instances of a single-unit on two successive days where the average waveform experienced subtle change while the ISIHs remained similar**.

On the other hand, all the ISIH-based dissimilarity measures did not provide the same degree of separation between classes that was provided by the peak matching. However, their inclusion as features was necessary to account for cases where the waveform shapes of two unit instances had subtle variability but their firing properties remain similar (see Figure 7).

### 3.2. Classifiers evaluation

**Figure 8:**
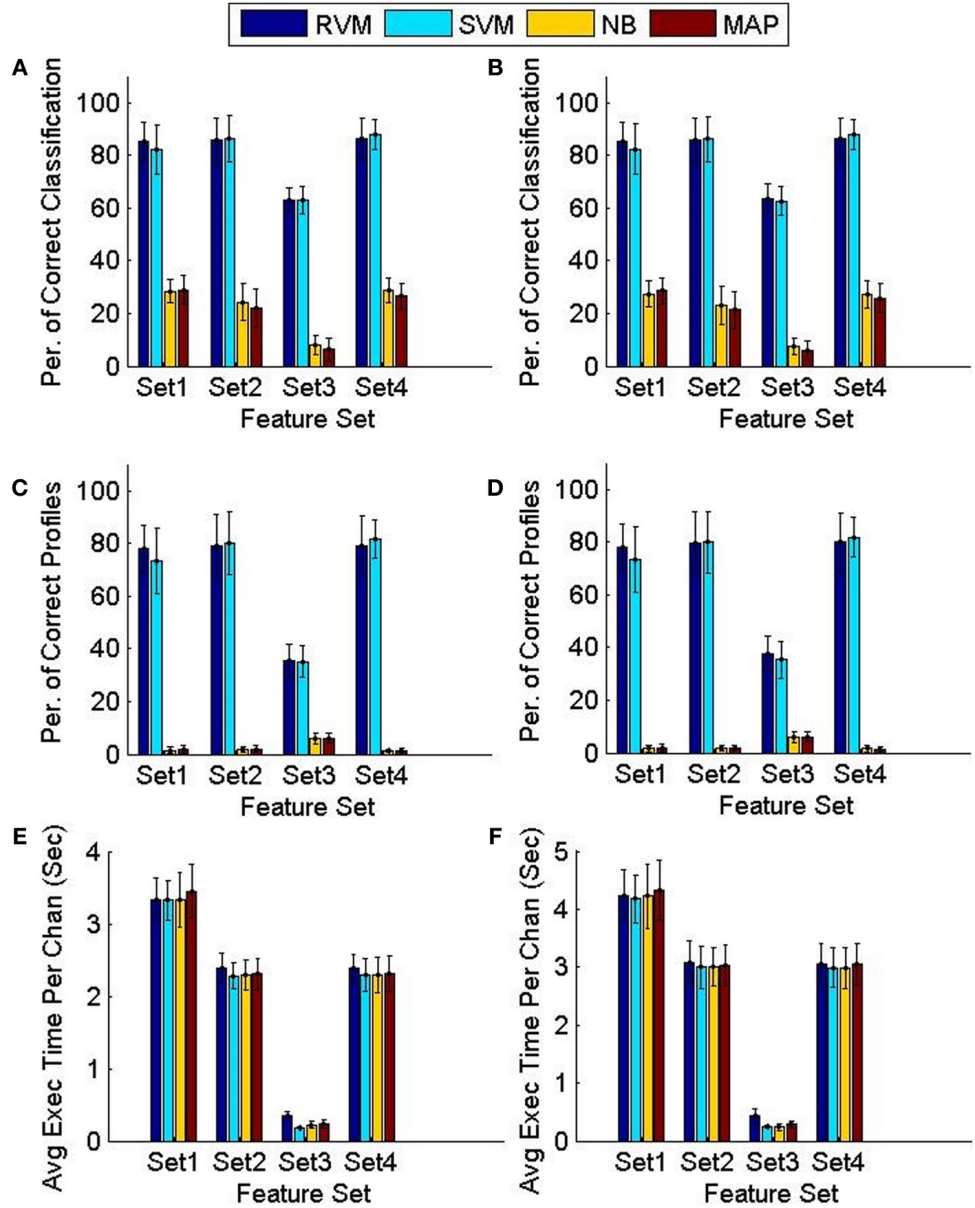
**Comparison of the classifiers' performance using the FMD and MMD methods**. Four different sets of features were used and the 6 validation datasets were used (each had 5 training datasets and 5 testing datasets). Error bars indicate the standard deviation (*n* = 6). Panels **(A,C,E)** show the performance of the FMD, while Panels **(B,D,F)** show the performance of the MMD. Using a Two-way ANOVA test, the RVM and SVM were found to perform significantly better than the NB and MAP (*p* < 0.01) and that feature set 3 is worse than all other feature sets (*p* < 0.01).

Figure 8 compares the tracking algorithm performance for the testing datasets using the four feature sets and using the two techniques proposed: FMD and MMD. A Two-way ANOVA test revealed effects by both the type of classifier used (*p* < 0.01) and the feature set used (*p* < 0.01). It is noticeable from Figure 8 that both the NB and MAP classifiers perform substantially worse than both the RVM and SVM classifiers. We performed another Two-way ANOVA test using **only** RVM and SVM data and it revealed an effect by the feature set used (*p* < 0.01) with no effect caused by the classifier used (*p* < 0.7). It is also noticeable from Figure 8 that feature sets 1,2, and 4 provide better tracking than feature set 3. Finally, we performed a Three-way ANOVA test to determine which tracking strategy achieves a higher performance (FMD or MMD). The test showed that both strategies performed very similarly (*p* < 0.95). Execution times were similar for different classifiers. Feature set 1 had the largest execution time, which is expected because of the larger number of features used.

**Figure 9:**
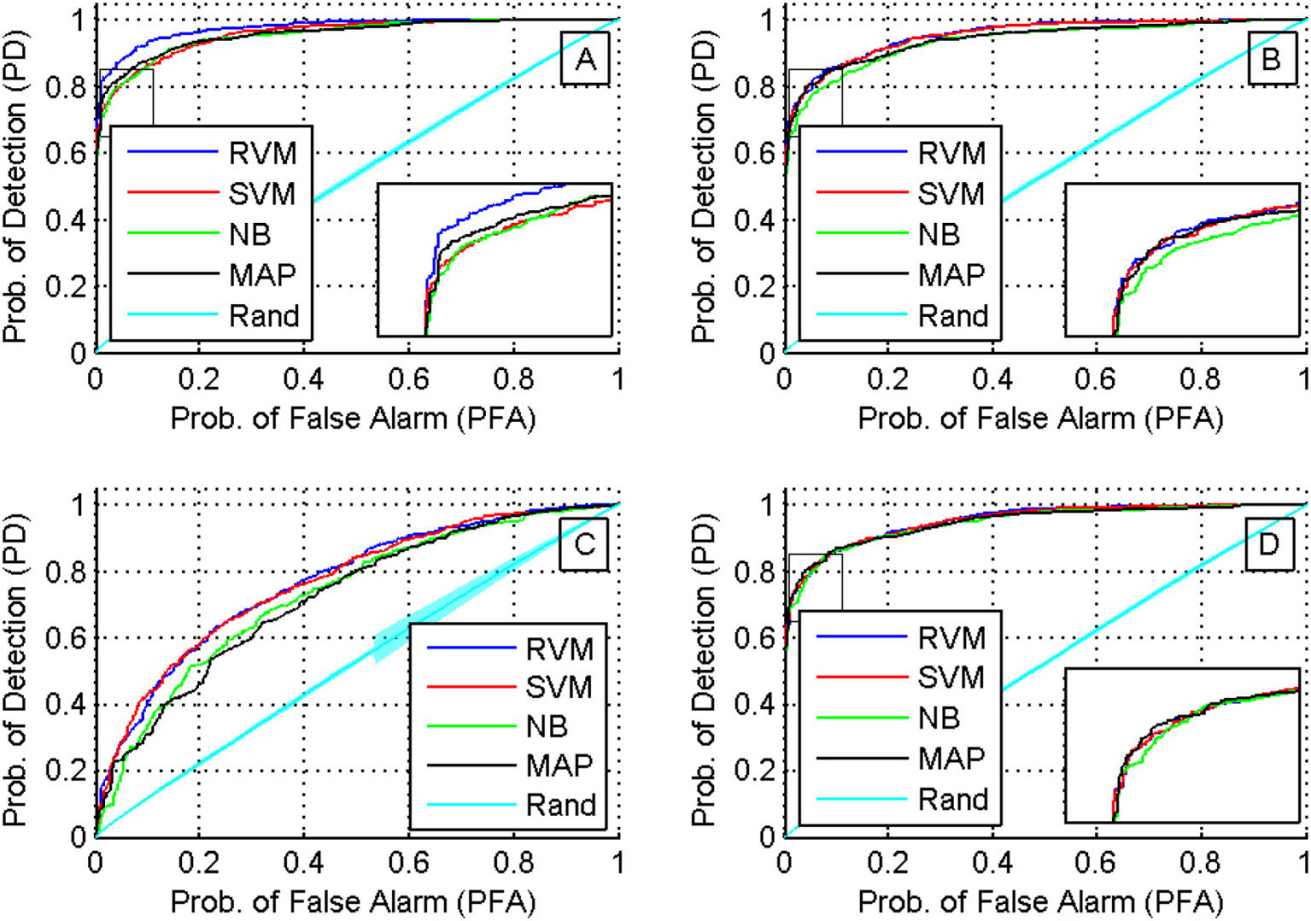
**ROC curves for different classifiers using different feature sets (sets 1 through 4 are shown in subfigures **(A–D)**, respectively)**. A Two-way ANOVA test on the AUC showed that there was no effect by the type of classifier used (*p* < 0.03).

We then compared the receiver operating characteristics (ROC) of the four classifiers by computing the area under the curve (AUC) when using each of the four feature sets. A Two-way ANOVA test showed that there was no effect by the type of classifier used (*p* < 0.03). Note that although there was a significant difference in the performance of the classifiers, they had similar ROC curves. The reason behind this is that the ROC curve measures only a classifier's performance in deciding whether two single-units are a possible match, while the calculation of the accuracy of the tracking algorithm measures also the performance of the additional back-tracking step post-processing. Figure 9 shows the ROC curves of the four classifiers using the four feature sets when trained on all seven training datasets. We also assessed the ROC for an RVM classifier trained by randomly shuffling the labels of the training datasets and repeating the process 100 times to establish chance level.

**Figure 10:**
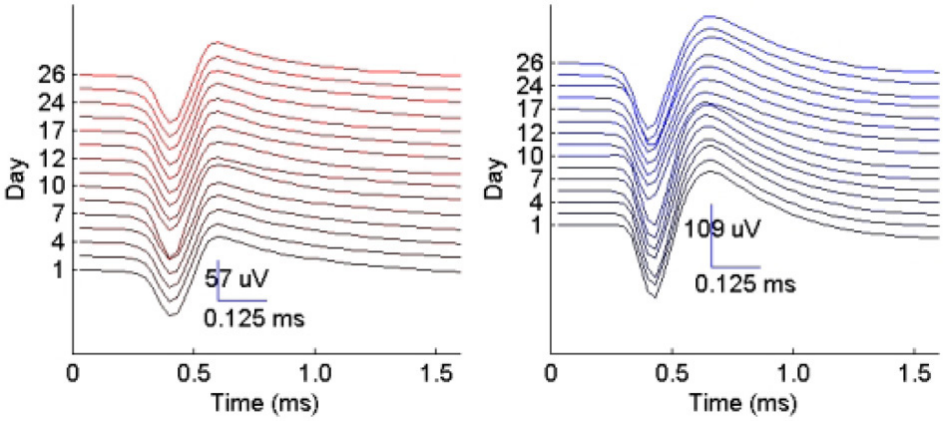
**Example of two single-unit profiles tracked by the RVM classifier across 15 datasets spanning a period of 26 days**.

We then examined the tracking algorithm performance when tested on additional datasets. Tables 3, 4 list the performance of the tracking algorithm using different classifiers in terms of percentage correct classification and percentage of correct profiles tracked when trained and tested using all 15 datasets described in section 2.6 (i.e., without partitioning). Similarly, the RVM and SVM classifiers performed much better than the NB and MAP classifiers. The RVM classifier was our choice for subsequent analysis due to its advantages of combining the SVM features with probabilistic classification. Figure 10 illustrates two single-unit profiles that were successfully tracked by the RVM classifier.

**Table 3:** **Comparison between the classifiers' performance using the Classification Accuracy when FMD and MMD were used**.

**Set/class**	**RVM**		**SVM**		**NB**		**MAP**	
**Strategy**	**FMD**	**MMD**	**FMD**	**MMD**	**FMD**	**MMD**	**FMD**	**MMD**
Set 1	90.51	91.67	87.44	88.05	51.26	50.98	50.85	52.28
Set 2	89.14	89.89	88.05	88.32	48.94	49.01	48.12	47.50
Set 3	61.16	62.11	61.92	67.84	22.45	22.45	16.65	16.99
Set 4	89.62	90.30	89.41	89.55	51.67	49.07	52.76	52.15

All of the 7 training datasets and 8 testing datasets were used to evaluate the classifiers over a longer duration of time. The results show that the performance didn't deteriorate significantly. MMD performed better than FMD in almost all the cases but with no statistical significance.

**Table 4:** **Comparison between the classifiers' performance using the Percentage of Correct Profiles when FMD and MMD were used**.

**Set/class**	**RVM**		**SVM**		**NB**		**MAP**	
**Strategy**	**FMD**	**MMD**	**FMD**	**MMD**	**FMD**	**MMD**	**FMD**	**MMD**
Set 1	78.34	82.67	73.62	74.80	2.75	3.14	1.96	2.36
Set 2	74.01	75.98	72.83	74.80	3.14	3.14	3.54	2.75
Set 3	20.47	26.37	15.74	29.92	5.90	5.11	5.51	5.90
Set 4	75.98	77.16	77.16	75.19	1.96	1.57	2.36	1.57

All of the 7 training datasets and 8 testing datasets were used to evaluate the classifiers over a longer duration of time. The results show that the performance did not deteriorate significantly. MMD performed better than FMD in almost all the cases but with no statistical significance.

We also found that the MMD approach had higher average execution time per channel than the FMD due to the additional number of comparisons the algorithm has to perform to find the closest matching unit within the “stability window.” On the other hand, Tables 3, 4 indicated that the MMD approach had slightly higher classification accuracy than the FMD because it was designed to find the closest match to the new unit. The MMD approach was therefore the choice for the remainder of the analysis.

We then investigated the trade-off between algorithm speed and accuracy (as listed in Tables 3–5). Feature set 1 achieved the highest performance but at the expense of longer execution time on average. On the other hand, feature set 4 had a shorter execution time than feature set 1 but at the expense of lower accuracy (second highest accuracy among the four feature sets). However, the benefit of using feature set 4 is greater than that of using feature set 1, since the drop in accuracy is negligible (1% drop) compared to the large increase in average execution time per channel (17% increase). Therefore, we used feature set 4 for further analysis.

**Table 5:** **Average execution time per channel when the RVM was used to track 15 datasets**.

Feature set	1	2	3	4
Time (s)	5.24	4.03	0.62	4.48

**Figure 11:**
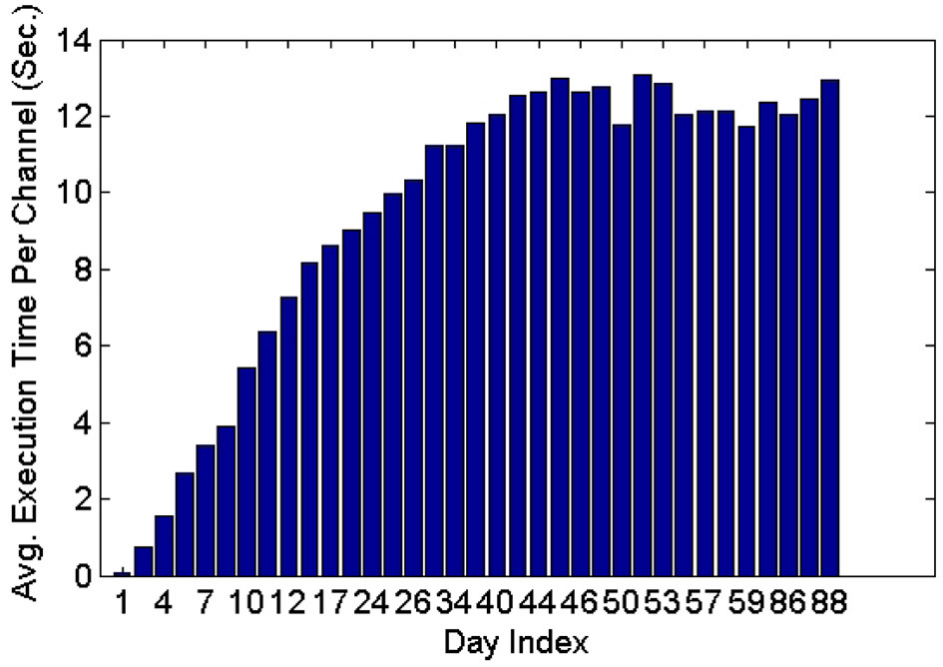
**Average execution time per channel when feature set 4, the RVM classifier and the MMD method were used**. The classifier was applied on 35 datasets spanning 88 days.

Figure 11 shows that the average execution time per channel increases as more datasets are included on subsequent days of recording until it reaches a plateau. This is expected because generally new units are unlikely to appear as time increases post implant, and thus the number of active profiles remains steady after the initial period has passed. Furthermore, the larger variation in the average execution time per channel in early recording sessions can be attributed to the lower number of comparisons made, which is in turn caused by the small number of instances in each profile and the small number of profiles created. Profiles inactive for a long time (>seven datasets) were dropped and were not included in the tracking.

## 4. Discussion

Stability of neuronal signals has been traditionally assessed by visual inspection of the similarity between units' waveforms to average waveforms from datasets collected across multiple days of recording (Rousche and Normann, 1998; Nicolelis et al., 2003; Carmena et al., 2005; Linderman et al., 2006; Chestek et al., 2007; Ganguly and Carmena, 2009). Few studies, however, have attempted to develop automated, quantitative solutions to the unit-stability tracking problem. Jackson and Fetz (2007) devised an approach to track units using the peak of the normalized cross-correlation between average spike waveforms. They reported that 80% of the stable units had correlation values of >0.95 across days. Suner et al. (2005) compared the signals recorded on the same channel across days by comparing the centers of clusters in the principal components space and used a Kolmogorov-Smirnov statistic between inter-spike intervals histograms of the formed clusters to ascertain their stability.

Tolias et al. (2007) determined the null distribution of pairwise distances between pairs of average waveforms by comparing signals before and after adjusting the depth of movable tetrodes. This distribution was then used to train a classifier that tracks the stability of single-units. The method, however, is inapplicable in the case of a fixed electrode array.

Dickey et al. (2009) trained a classifier on features extracted from both the unit's average waveform and ISIH. The authors argued that using both the average waveforms and ISIHs resulted in improved accuracy. However, they noted that the use of the ISIH as a stability criterion may not apply when learning is taking place. Furthermore, their method of comparing ISIHs is computationally prohibitive, which makes it unfavorable for BMI applications where the assessment of the single-unit stability from short-duration neural recordings is ultimately desired.

Fraser and Schwartz (2012) used features extracted from functional relations between units, such as pairwise cross correlograms, to track units across days. This method, however, requires the subject to be engaged in a behavioral task and may be susceptible to plasticity-mediated changes in functional connectivity between units as learning progresses.

In this study, we presented an algorithm for automated tracking of multiple single units across many days that addresses most of the aforementioned problems. In particular, it is applicable to recordings from high-channel count microelectrode arrays. Notably, the algorithm requires short-duration neural recordings (15 min) without the need for the subject to be engaged in any behavioral task. The algorithm is not computationally intensive and, therefore, applicable in real time BMI applications.

We compared the tracking algorithm accuracy when four classifiers were used: RVM, SVM, MAP, and NB. Both RVM and SVM were superior to MAP and NB and there were no statistically significant differences between their results. We recommend the use of RVM since it combines the advantages of SVM with the notion of a probabilistic classifier and in our experience it constantly provided good tracking results.

We also compared model performance across four different cases of input feature sets based on waveform features and firing characteristics. We also proposed a technique to boost the performance of the tracking algorithm by comparing newly recorded single-units to multiple instances of a putative single-unit from its stored profile across days and selecting the closest instance to the new unit. When the algorithm was tested on eight datasets (corresponding to 16 consecutive calendar-days), this strategy resulted in a classification accuracy of ~91% when using all the features (feature set 1), and ~90% when using only ~37% of the features (feature set 4). Additionally, 82 and 77% of the profiles tracked by the algorithm matched the experts' manual tracking using feature set 1 and feature set 4, respectively. The average execution time was short and converged to ~12 s per channel after ~45 data sets (with feature set 1 in use), though can be further reduced to ~8 s per channel at a slightly lower accuracy (with feature set 4 in use). Thus, as more recordings accumulate across days, the process gets less demanding and can be executed more rapidly. The poorest performance was obtained when we used the ISIH-based features only. The results show that adding the ISIH-based features can help improve the performance but cannot successfully track single units on their own. This can be explained by the fact that subjects were not over-trained on the Brain-control task, and thus single units' firing characteristics were largely variable during the learning phase (Hikosaka et al., 2002). Given the speed-accuracy tradeoff, we suggest using feature set 4 which includes a subset of waveform-based dissimilarity measures: normalized peak-to-peak height difference, normalized peak-to-peak time difference, and peak matching. In addition, using feature set 4 minimizes the adverse effect that variable ISIHs may have on performance.

Our algorithm is based on one generic classifier that is used for all channels. However, different channels may have different characteristics. For example, units may vary on one (or more) channel more than others. A human expert can capture the extent of variability of a specific channel across days, while an automated method cannot because some training samples might be outliers. One possible improvement in such case is to perform a two-level classification paradigm. In this paradigm, a custom classifier for each channel is trained using features extracted from this channel's units only.

It is noteworthy that the single-unit stability tracking problem can be postulated as a Graph Matching or a Graph Cut problem (Cormen et al., 2001). In particular, a node in the graph would represent each single-unit occurrence on a given day, and the edges between nodes would represent the dissimilarity measure between the single-units appearing on two different days. A graph matching procedure that matches single-units across two different days is equivalent to Maximum Bipartite Graph Matching (Cormen et al., 2001). Generalizing the tracking problem to more than two recordings requires solving a Graph Partition problem which generally falls in the category of NP-hard problems (Bichot and Siarry, 2013). The algorithm provided in this paper is a fast approximate solution to the single-unit tracking problem.

We note that our algorithm performance was based on spike data sorted online using the spike sorter tool in the Cerebus® software (Blackrock microsystems, UT). Online spike sorters that use the “hoops” method for labeling spikes based on time and amplitude features of the waveforms perform poorer than offline spike sorting algorithms that use more sophisticated feature extraction and clustering techniques, since spike-amplitude instability is known to cause spike detection and sorting errors for such methods (Perge et al., 2013). As such, it is expected that more sophisticated online spike detection (Oweiss, 2010) and sorting techniques (Aghagolzadeh and Oweiss, 2009), particularly those that permit efficient tracking using simple methods (Aghagolzadeh et al., 2013) would result in an overall performance gain compared to the traditional methods implemented in commercially available systems.

The proposed technique may also improve the clinical viability of BMI systems in a patient's home settings. If single units can be autonomously, accurately and rapidly tracked across days without much supervision from a caregiver, this would ease its use, and would facilitate maintaining a fixed mapping between the neural input space and the decoded output, which may likely increase the utility of the BMIs over the long term.

Finally, the application of the technique may extend well beyond ensuring that a stable BMI is maintained over days. In particular, studies of learning that aim to elucidate neural mechanisms of plasticity at the cellular and population levels *in vivo* can significantly benefit from the proposed approach. These studies, however, are in their infancy, primarily due to the inability to validate unit identity in intermittent extracellular recording sessions across multiple days. As such, it is often assumed that units recorded on a given day are not the same as the ones recorded on other days, which “artificially inflates” the unit sample size. With technological advances that lead to ever increasing number of electrodes, the approach provides a novel framework to streamline the process over consecutive days that can help assess learning-induced plasticity in local and distributed neural circuits.

In conclusion, we proposed a technique that could substantially alleviate the burden on the caregiver as well as the patient in a clinical BMI setting. By automating the tracking of multiple single-units across many days of recordings from high-channel-count microelectrode arrays, we're essentially maximizing the likelihood of maintaining a fixed mapping between the neural input space and the decoded output, which in turn may accelerate BMI learning and reduce the cognitive load on the patient side. This may improve the clinical viability and rapid adoption of BMI systems in home settings by end users.

### Conflict of interest statement

The authors declare that the research was conducted in the absence of any commercial or financial relationships that could be construed as a potential conflict of interest.
